# Insulin Bidirectionally Alters NAc Glutamatergic Transmission: Interactions between Insulin Receptor Activation, Endogenous Opioids, and Glutamate Release

**DOI:** 10.1523/JNEUROSCI.3216-18.2021

**Published:** 2021-03-17

**Authors:** Tracy L. Fetterly, Max F. Oginsky, Allison M. Nieto, Yanaira Alonso-Caraballo, Zuleirys Santana-Rodriguez, Carrie R. Ferrario

**Affiliations:** ^1^Department of Pharmacology, University of Michigan, Ann Arbor, Michigan 48109; ^2^Department of Psychiatry, Harvard Medical School, McLean Hospital, Belmont, Massachusetts 02478

**Keywords:** glutamate, insulin receptor, motivation, nucleus accumbens, opioid, striatal plasticity

## Abstract

Human fMRI studies show that insulin influences brain activity in regions that mediate reward and motivation, including the nucleus accumbens (NAc). Insulin receptors are expressed by NAc medium spiny neurons (MSNs), and studies of cultured cortical and hippocampal neurons suggest that insulin influences excitatory transmission via presynaptic and postsynaptic mechanisms. However, nothing is known about how insulin influences excitatory transmission in the NAc. Furthermore, insulin dysregulation accompanying obesity is linked to cognitive decline, depression, anxiety, and altered motivation that rely on NAc excitatory transmission. Using whole-cell patch-clamp and biochemical approaches, we determined how insulin affects NAc glutamatergic transmission in nonobese and obese male rats and the underlying mechanisms. We find that there are concentration-dependent, bidirectional effects of insulin on excitatory transmission, with insulin receptor activation increasing and IGF receptor activation decreasing NAc excitatory transmission. Increases in excitatory transmission were mediated by activation of postsynaptic insulin receptors located on MSNs. However, this effect was due to an increase in presynaptic glutamate release. This suggested feedback from MSNs to presynaptic terminals. In additional experiments, we found that insulin-induced increases in presynaptic glutamate release are mediated by opioid receptor-dependent disinhibition. Furthermore, obesity resulted in a loss of insulin receptor-mediated increases in excitatory transmission and a reduction in NAc insulin receptor surface expression, while preserving reductions in transmission mediated by IGF receptors. These results provide the first insights into how insulin influences excitatory transmission in the adult brain, and evidence for a previously unidentified form of opioid receptor-dependent disinhibition of NAc glutamatergic transmission.

**SIGNIFICANCE STATEMENT** Data here provide the first insights into how insulin influences excitatory transmission in the adult brain, and identify previously unknown interactions between insulin receptor activation, opioids, and glutamatergic transmission. These data contribute to our fundamental understanding of insulin's influence on brain motivational systems and have implications for the use of insulin as a cognitive enhancer and for targeting of insulin receptors and IGF receptors to alter motivation.

## Abstract (precis)

Human fMRI studies show that insulin influences brain activity in regions that mediate reward and motivation, including the nucleus accumbens (NAc). Insulin receptors are expressed by NAc medium spiny neurons (MSNs), and studies of cultured cortical and hippocampal neurons suggest that insulin influences excitatory transmission via presynaptic and postsynaptic mechanisms. However, nothing is known about how insulin influences excitatory transmission in the NAc.

## Introduction

Recent studies in humans suggest that insulin may enhance cognition and decision-making processes that influence reward-seeking (Reger et al., 2008; Freiherr et al., 2013). In addition, actions of insulin within mesocorticolimbic circuits influence hunger, motivation, and feeding behaviors (Liu and Borgland, 2015; Woods et al., 2016; Ferrario and Reagan, 2018). However, the mechanisms by which insulin affects neural function in the adult brain are poorly understood (Biessels and Reagan, 2015; Ferrario and Reagan, 2018). Studies in cortical and hippocampal neurons have shown that insulin influences excitatory transmission via presynaptic mechanisms that reduce glutamate release, as well as postsynaptic mechanisms that affect AMPAR trafficking (Beattie et al., 2000; Man et al., 2000; Passafaro et al., 2001; Huang et al., 2004; Labouebe et al., 2013; Liu et al., 2013). However, these studies were conducted in cultured neurons or juvenile rodents (18-30 d old). Thus, very little is known about effects of insulin on excitatory transmission in the adult brain.

Glutamatergic transmission within the NAc mediates many aspects of motivation and decision-making in response to food, sex, and drugs of abuse, as well as to environmental stimuli paired with these rewards. For example, food- and drug-seeking behaviors rely on activation of the NAc (Di Ciano et al., 2001; Kalivas, 2009; Wolf, 2016), and repeated exposure to drugs of abuse or palatable foods enhances NAc excitatory transmission that underlies food- and drug-seeking behaviors (Oginsky et al., 2016; Wolf, 2016; Dong et al., 2017; Derman and Ferrario, 2018; Alonso-Caraballo et al., 2020; Ferrario, 2020). Thus, identifying neural mechanisms that regulate NAc excitatory transmission is fundamental to understanding the neurobiology of normal and aberrant motivation.

Here, we used whole-cell patch clamp recordings in adult rat brain slices to determine how insulin affects excitatory transmission onto NAc medium spiny neurons (MSNs) and the mechanisms involved. Importantly, in addition to insulin receptors, insulin-like growth factor receptors (IGFRs) are also expressed in the NAc and can be activated by moderate to high concentrations of insulin (Unger et al., 1989; Schumacher et al., 1991). Thus, a wide range of insulin concentrations were examined, and the contribution of insulin receptor activation versus IGFR activation to insulin's effects were determined. In addition, given that obesity is associated with insulin dysregulation, altered NAc excitatory transmission, cognitive deficits, and some psychiatric diseases (Biessels and Reagan, 2015; Kullmann et al., 2016; Stoeckel et al., 2016), we also determined how high-fat diet-induced obesity alters insulin's ability to influence NAc excitatory transmission.

We found that insulin receptor and IGFR activation have opposing effects on excitatory transmission in the NAc, with insulin receptor activation increasing, and IGFR activation decreasing, presynaptic glutamate release. Furthermore, insulin-induced increases in glutamate release occurred through a previously unidentified opioid receptor-dependent disinhibition that relied on GABA_B_-receptor activation. Finally, diet-induced obesity resulted in a loss of insulin-induced increases in NAc excitatory transmission and a reduction in NAc insulin receptor surface expression. Together, these data reveal novel roles for insulin in the regulation of NAc excitatory transmission, provide new insights into opioid-dependent regulation of NAc glutamatergic transmission, and have implications for endogenous and exogenous insulin in modulating motivation and reward.

## Materials and Methods

### 

#### Animals

Male Sprague Dawley rats were purchased from Envigo, pair-housed (reverse light-dark; 12/12, lights off at 7:00 A.M.) with free access to food and water unless otherwise stated (70-80 d old). All procedures were approved by the University of Michigan Institutional Animal Care & Use Committee. For additional information, see also https://sites.google.com/a/umich.edu/ferrario-lab-public-protocols/.

#### Electrophysiology

Whole-cell patch-clamp recordings of MSN in the NAc core were conducted as previously described (Ferrario et al., 2011; Oginsky et al., 2016). Rats were anesthetized with chloral hydrate (400 mg/kg, i.p.; slices prepared between 10:00 and 11:00 A.M.), brains were rapidly removed and placed in ice-cold oxygenated (95% O_2_-5% CO_2_) aCSF containing the following (in mm): 125 NaCl, 25 NaHCO_3_, 12.5 glucose, 1.25 NaH_2_PO_4_, 3.5 KCl, 1 L-ascorbic acid, 0.5 CaCl_2_, 3 MgCl_2_, 295-305 mOsm, pH 7.4. Coronal slices (300 μm) containing the NAc were made using a vibratory microtome (Leica Biosystems) and allowed to rest in oxygenated aCSF (40 min). For the recording aCSF (2 ml/min), CaCl_2_ was increased to 2.5 mm and MgCl_2_ was decreased to 1 mm. Patch pipettes were pulled from 1.5 mm borosilicate glass capillaries (WPI; 3-7 mΩ resistance) and filled with a solution containing the following (in mm): 140 CsCl, 10 HEPES, 2 MgCl_2_, 5 Na^+^-ATP, 0.6 Na^+^-GTP, 2 QX314, pH 7.3, 285 mOsm. All recordings were conducted in the presence of picrotoxin (50 μm) to isolate excitatory transmission. Evoked EPSCs (eEPSCs) were elicited by local stimulation (0.05-0.30 mA square pulses, 0.3 ms, delivered every 20 s) using a bipolar electrode placed ∼300 μm lateral to recorded neurons. The minimum amount of current needed to elicit a synaptic response with <15% variability in amplitude was used. If >0.30 mA was required, the neuron was discarded. eEPSCs were recorded at −70 mV. Baseline (BL) responses were established (10 min) followed by a bath application of insulin in the presence or absence of antagonists (10 min). Miniature EPSCs (mEPSCs) were recorded in the presence of TTX (1 μm) at a holding potential of –65 mV. To validate the paired-pulse facilitation procedure, eEPSCs were measured across a range of interpulse intervals (50, 75, 100, 200, and 400 ms; 6-8 pulses per interval) in a set of control cells. Facilitation was reliably produced at an interval of 50 ms; thus, this interval was used in our experiments. The probability of glutamate release was determined by dividing the averaged amplitude of the second peak by the averaged amplitude of the first peak (i.e., paired-pulse ratio [PPR]). Recorded signals were amplified with a Multiclamp 700B (Molecular Devices), digitized at 20 kHz, and filtered at 2 kHz and collected with Clampex 10.4 data acquisition software (Molecular Devices). All drugs were bath-applied for 10 min (Sigma Millipore: insulin [91077C], phaclofen [114012-12-3], (–)-naloxone [51 481-60-8], bestatin [65 391-42-6], thiorphan [76721-89-6]), HNMPA and HNMPA-(AM)3 (Santa Cruz Biotechnology; sc-205714, sc-221730), picropodophyllotoxin (PPP, Tocris Bioscience, catalog # 2956), (+)-naloxone was provided by Kenner C. Rice (Drug Design and Synthesis Section, National Institute on Drug Abuse Intramural Research Program). In our initial studies, insulin concentrations ranging from 1 to 500 nm were used. This was done in part to facilitate comparison to effects of insulin in other brain regions, including the VTA where concentrations of 100 and 500 nm have been used (Labouebe et al., 2013; Liu et al., 2013) and to avoid missing effects by examining just one concentration. Furthermore, while physiological concentrations of insulin are thought to be relatively low (∼10-30 nm), how these levels may be affected by diet-induced obesity and/or the diabetic state is not understood; therefore, levels could be much higher (for additional discussion, see Ferrario and Reagan, 2018).

#### Single-cell RT-PCR and identification of D1- and D2-type MSNs

Single-cell RT-PCR was conducted on cell contents taken from MSNs after whole-cell recordings to identify D1- and D2-type MSNs. The first-strand cDNA synthesis was performed using the Superscript III First-Strand Synthesis System for RT-PCR (Invitrogen) per the manufacturer's instructions. The reverse transcription product was kept at −20°C until PCR was performed. PCR primers used are as follows: prodynorphin forward: 5′-GCCTAGGAGTGGAGTGTTCG, reverse: 5′- GGGATAGAGCAGTTGGGCTG; proenkephalin forward: 5′- ATGCCATGCCATCGGGAAG, reverse: 5′- CAGGACCAGCAGGGACAATC. PCR product lengths were >100 bp so as to not confuse them with primer dimers; 4 μl (prodynorphin) or 6 μl (proenkephalin) of reverse transcription product was loaded into an Eppendorf tube with PCR solution containing 10 μl of 5× green GoTaq flexi buffer, 2 μl MgCl_2_, 1 μl of 10 mm dNTP mix, 1 μl of 10 mm forward and reverse primers, 0.25 μl of GoTaq polymerase (Promega), and brought up to a final volume of 50 μl with nuclease-free water. The thermal cycling program was set to the initial denaturation for 5 min at 95°C for one cycle. The denaturation, annealing, extension cycles were done at 95°C for 1 min, 58°C (pENK) and 65°C (pDYN) for 1 min, and 72°C for 1 min, respectively, for 45 cycles. A final extension cycle was done at 72°C for 5 min; 4 μl of the PCR was placed into a second PCR tube with the same solution as before, and the same cycling protocol was performed; 20 μl from the second PCR was run on a 2% agarose gel containing ethidium bromide. Gels were imaged using GelDoc-It^2^ imager (UVP). D1- or D2-type MSNs were defined by the presence of a PCR product band for either prodynorphin or proenkephalin, respectively.

#### High-fat diet-induced obesity

Rats were given free access to 60% high-fat diet (Open Source Diets D12492) in the home cage for a total of 8 weeks. Controls had free access to standard lab chow throughout (Lab Diet 5001, 13% fat). Weight was measured twice each week. In addition, after 7 weeks of high-fat or control diet, body composition was determined by NMR (Minispec LF90II, Bruker Optics), and fasted blood samples (16 h) were collected and used to determine plasma insulin levels. Blood samples were collected via tail nick into tubes containing EDTA (1.6 mg/ml, Sarstedt), and plasma was then isolated by centrifugation (1000 × *g*, 4°C, 10 min) and stored (−20°C) for subsequent analysis as previously described (Vollbrecht et al., 2015). Plasma insulin levels were determined by double-antibody radioimmunoassay using a ^125^I-human insulin tracer (Linco Research), a rat insulin standard (Novo Nordisk), a guinea pig anti-rat insulin first antibody (Linco Research), and a sheep anti-guinea pig γ globulin-PEG second antibody (Michigan Diabetes Research Core). Blood collection and NMR were conducted at week 7 to avoid additional stress during the week of slice preparation or NAc tissue collection (week 8). Food was removed from the cage 1-2 h before slice preparation or NAc tissue collection.

#### Biochemistry

##### Purification of surface (bound) proteins, and Western blotting

NAc tissue was biotinylated and NeutrAvidin isolation of biotinylated (surface) proteins was conducted as previously described (Ferrario et al., 2011). For these experiments, verification studies were done to determine optimal pulldown procedures and the amount of material to be loaded per lane. Briefly, bilateral NAc tissue (containing core and shell) from each rat was dissected and chopped (400 μm; McIllwain tissue chopper; Vibratome). NAc tissue was added to ice-cold aCSF containing 1 mm sulfo-NHS-S-S-Biotin (Thermo Fisher Scientific) and incubated with gentle agitation (30 min, 4°C). This reaction was quenched by the addition of glycine (100 mm, 10 min, 4°C), tissue was pelleted, and resuspended in ice-cold lysis buffer (in mm as follows: 25 HEPES, 500 NaCl, 2 EDTA, 1 PMSF, 20 NaF, 1:100 protease inhibitor cocktail set I [Calbiochem], and 0.1% Nonidet P-40 [v/v]; pH 7.4), sonicated and stored at −80°C for subsequent use.

Procedures to purify biotinylated (i.e., surface) proteins were adapted from Thermo Fisher Scientific product instructions, and all steps were conducted on ice or at 4°C unless otherwise noted. Protein concentrations were determined by Pierce BCA assay; 100 µg of NAc protein was added to high-capacity NeutrAvidin agarose beads (Thermo Fisher Scientific, catalog #29202) and incubated overnight with end-over-end rotation. Biotinylated proteins bound to NeutrAvidin beads (bound, surface fraction) were isolated from the nonbiotinylated (unbound) fraction by centrifugation (3000 RPM, 1 min) and washed (3 times, 1 × PBS). The supernatant (unbound) was collected, and fresh beads were added for a second overnight incubation and isolation of surface proteins as above. The bound fractions were combined in a total of 70 µl of Laemmli sample treatment buffer containing DTT (100 mm), and heated at 97°C for 3 min to release the biotinylated proteins from the beads. The bound samples were then spun at 10 000 RPM for 5 min on a centrifugal filter unit (0.45 mm, #UFC30HV00, Millipore) to remove the NeutrAvidin beads from the solution. The samples were then stored at −20°C until used for Western blotting.

For Western blotting, bound fractions (surface protein) or whole-cell lysates (total protein) were heated (70°C, 10 min), loaded into gels (20 µg whole cell lysate, 20 µl bound fraction), and electrophoresed under reducing conditions. Proteins were transferred onto PVDF membranes (Millipore, catalog #IPVH00010), membranes were rinsed, blocked (1 h, room temperature, 5% [w/v] nonfat dry milk in TBS-Tween 20 [TBS-T; 0.05% Tween 20, v/v]), and incubated overnight with primary antibody to the β subunit of the insulin receptor (IRβ; 1:200 in TBS; Santa Cruz Biotechnology, S711). To verify that intracellular proteins were not present in the bound fraction, the relative expression of TH (1:30,000 in TBS; Invitrogen, P21962) was determined in the bound and unbound fractions. Membranes were then washed in TBS-T, incubated with HRP-conjugated secondary (Invitrogen; 1 h, room temperature), washed, and immersed in chemiluminescence detecting substrate (GE Healthcare). Images were acquired on film, and Ponceau S (Sigma Millipore) was used to determine total protein in each lane. Bands of interest were quantified using ImageJ (National Institutes of Health).

#### Experimental design and statistical analysis

eEPSCs were analyzed with Clampfit 10.4 (Molecular Devices), mEPSCs were analyzed using Mini Analysis program 6.0.4 (Synaptosoft) and verified by hand. No more than 3 cells were included/rat for any given measure to avoid over-representation of 1 subject. Two-tailed *t* tests, one-way or two-way repeated measures ANOVAs, and Sidak's *post hoc* multiple comparisons tests were conducted using Prism 6-8 software (GraphPad). Statistical tests used for each data set are stated in Results and in brief in the figure legends. *N* values are given in Results, with the number of cells followed by the number of rats used for electrophysiological recordings (e.g., 6,5 = 6 cells from 5 rats).

## Results

### Insulin bidirectionally influences NAc excitatory transmission

**Figure 1.**
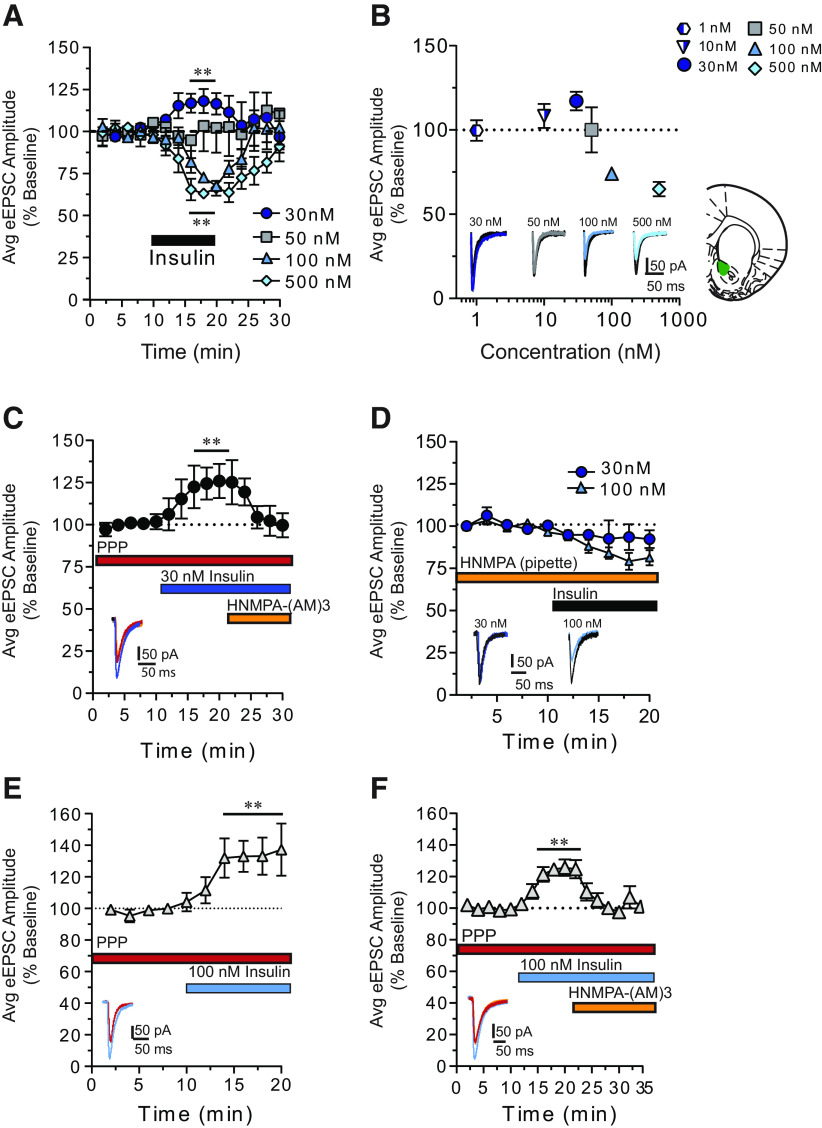
Insulin receptor activation increases, whereas IGFR activation decreases, excitatory transmission onto MSNs in the NAc core. ***A***, Average eEPSC amplitude during baseline (BL), bath application of insulin (black bar), and following washout. ***B***, Summary of average maximum change from BL following insulin (1-500 nm). Effects of insulin on excitatory transmission are concentration-dependent and bidirectional. Right, Recording location within the NAc core. ***C***, Average eEPSC amplitude in the presence of the IGFR antagonist PPP, before and after 30 nm insulin, with and without the membrane-permeable insulin receptor inhibitor HNMPA-(AM)3. ***D***, Average eEPSC amplitude before and after insulin (30 and 100 nm) with the membrane-impermeable insulin receptor inhibitor HNMPA included in the patch pipette. ***E***, Average eEPSC amplitude before and after 100 nm insulin administered in the presence of PPP. ***F***, Average eEPSC amplitude in the presence of PPP, before and after 100 nm insulin followed by the addition of HNMPA-(AM)3 to the bath. Data in all figures are shown as the mean ± SEM. Statistical differences were determined by within-subject, two-way repeated-measures ANOVA comparing BL and treatment conditions. **p < 0.01, main effect of treatment (for full statistical information, see Results).

Using whole-cell patch-clamping approaches from adult brain slices (Ferrario et al., 2011; Oginsky et al., 2016), we first determined how bath application of insulin (1-500 nm) affects the amplitude of eEPSCs in MSNs of the NAc core (Fig. 1). We found that 30 nm insulin significantly increased eEPSC amplitude (Fig. 1*A*, closed circles; two-way repeated-measures ANOVA main effect 30 nm: *F*_(1,7)_ = 10.55, *p* = 0.01; *N* = 7,6), whereas 100 or 500 nm insulin produced a significant decrease in amplitude (Fig. 1*A*, triangles, diamonds; two-way repeated-measures ANOVA main effect 100 nm: *F*_(1,4)_ = 19.56, *p* = 0.01; *N* = 5,4; main effect 500 nm: *F*_(1,4)_ = 43.50, *p* = 0.003; *N* = 5,4). eEPSC amplitude returned to BL following insulin washout. Furthermore, eEPSC amplitude was unchanged by 50 nm (Fig. 1*A*, squares; two-way repeated-measures ANOVA main effect 50 nm: *F*_(1,3)_ > 0.000006, *p* = 0.99; *N* = 4,3), 1 nm or 10 nm insulin (Fig. 1*B*, heptagon, triangle; two-way repeated-measures ANOVA main effect 1 nm: *F*_(1,5)_ = 0.093, *p* = 0.77; *N* = 6,4; main effect 10 nm: *F*_(1,3)_ = 1.617, *p* = 0.29; *N* = 4,2). Thus, insulin produces bidirectional and concentration-dependent effects on NAc excitatory transmission.

### Insulin receptor and IGFR activation have opposing effects on NAc excitatory transmission

In the adult brain, insulin activates insulin receptors and IGFRs (Vigneri et al., 2010). However, because of the different binding affinities of these receptors, low concentrations of insulin (∼30 nm) preferentially activate insulin receptors, whereas higher concentrations also activate IGFRs (Schumacher et al., 1991). We therefore hypothesized that increases in excitatory transmission elicited by 30 nm insulin may be mediated by insulin receptors, whereas decreases following 100 nm may be mediated by IGFRs. To test this, we applied selective antagonists of the IGFR (PPP, 500 nm) (Labouebe et al., 2013) or the insulin receptor blocker HNMPA-(AM)3 (100 μm) (Saperstein et al., 1989; Mebel et al., 2012) before or after insulin (Fig. 1*C–F*). Additional controls were conducted to assess the effect of these drugs on BL eEPSC amplitude. PPP increased eEPSC amplitude on its own (data not shown; two-way repeated-measures ANOVA main effect PPP: *F*_(1,8)_ = 24.0, *p* = 0.001; *N* = 5,3). Therefore, PPP was always applied before additional drug manipulations to allow for a stable BL to be established. Application of HNMPA-(AM)3 to the bath alone did not alter eEPSC amplitude (data not shown; two-way repeated-measures ANOVA main effect HNMPA-(AM)3: *F*_(1,3)_ = 0.004, *p* = 0.95; *N* = 4,3). Application of 30 nm insulin in the presence of the IGFR antagonist PPP resulted in a significant increase in eEPSC amplitude that was reversed by the subsequent addition of the membrane-permeable insulin receptor blocker, HNMPA-(AM)3 (Fig. 1*C*; two-way repeated-measures ANOVA condition × time interaction: *F*_(8,48)_ = 2.91, *p* = 0.01; *N* = 7,4). Furthermore, when the membrane-impermeable insulin receptor blocker HNMPA (300 μm) (Baltensperger et al., 1992; Labouebe et al., 2013) was included in the recording pipette, 30 nm insulin-induced increases in eEPSC amplitude were completely blocked (Fig. 1*D*, circles; two-way repeated-measures ANOVA: *F*_(1,4)_ = 2.7, *p* = 0.17; *N* = 5,4), while 100 nm insulin-induced decreases in eEPSC amplitude were still observed (Fig. 1*D*, triangles; two-way repeated-measures ANOVA main effect 100 nm insulin: *F*_(1,8)_ = 19.2, *p* = 0.002; *N* = 9,5). As this manipulation would only prevent activation of insulin receptors within the recorded MSN, these data indicate that increases in excitatory transmission are due to activation of insulin receptors located on MSNs. This effect could be mediated by increases in postsynaptic glutamate transmission, or to increases in glutamate release due to feedback from MSNs to presynaptic terminals.

When the IGFR antagonist PPP was applied before 100 nm insulin, previously observed decreases in eEPSC amplitude were absent (Fig. 1*E*). Furthermore, under this condition, insulin produced a significant increase in eEPSC amplitude (Fig. 1*E*; two-way repeated-measures ANOVA main effect 100 nm + PPP: *F*_(1,5)_ = 7.55, *p* = 0.04; *N* = 6,4), likely due to activation of insulin receptors (which are not blocked by PPP). To verify this, PPP was included in the bath followed by 100 nm insulin with and without HNMPA-(AM)3. Under these conditions, insulin-induced increases were completely reversed by the insulin receptor blocker (Fig. 1*F*; two-way repeated-measures ANOVA main effect of condition: *F*_(2,16)_ = 12.5, *p* = 0.0005; *N* = 9,5). Together, these data demonstrate that insulin receptors and IGFRs work in opposition to enhance and reduce NAc excitatory transmission, respectively.

### Identification of D1- and D2-type MSNs after whole-cell recording

**Figure 2.**
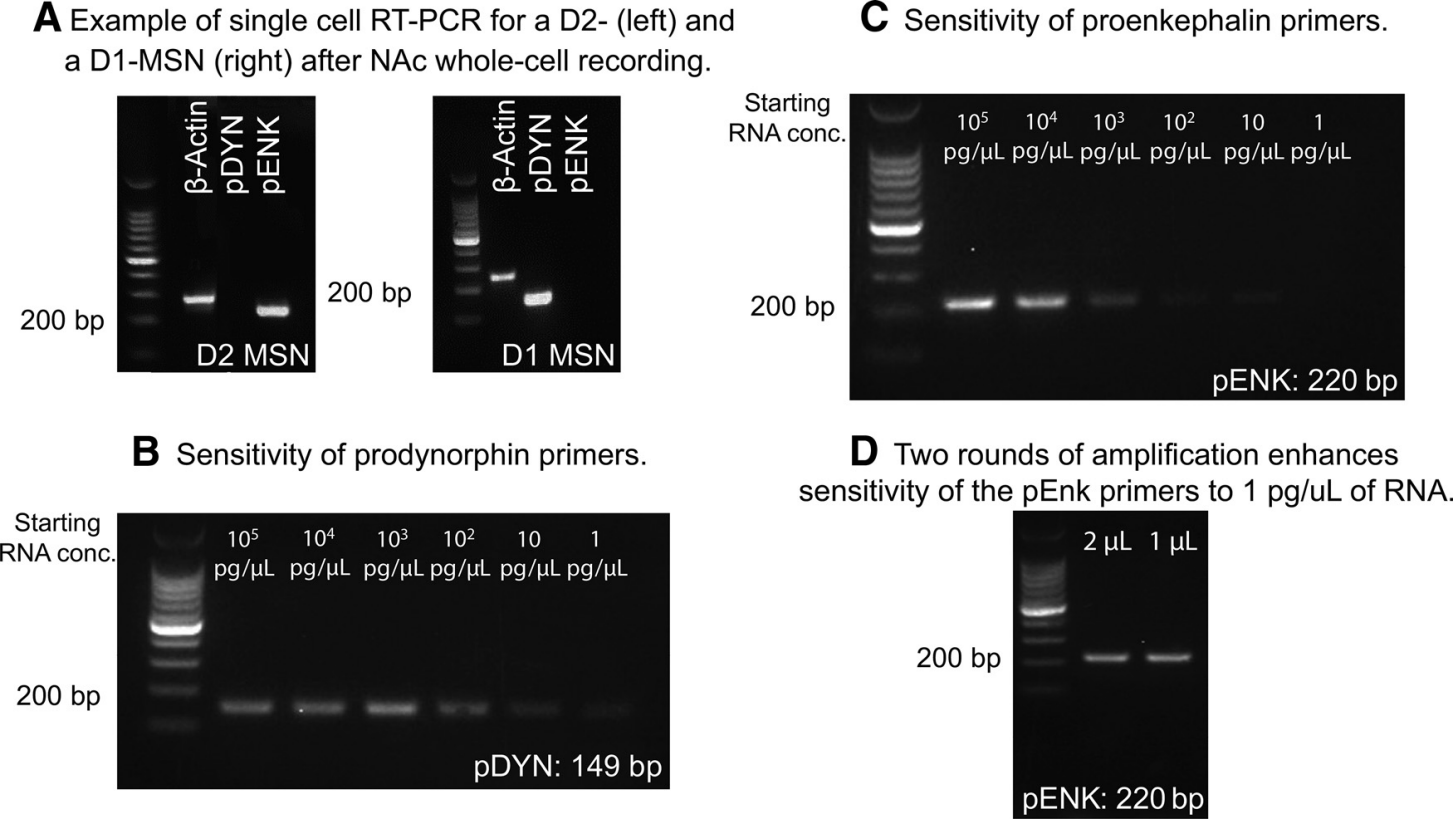
Verification of single-cell RT-PCR method. ***A***, Example of single-cell RT-PCR for a D2-MSN (left) and a D1-MSN (right) after whole-cell recordings in adult rat nucleus accumbens. β-actin was used as a positive control. ***B***, Serial dilution of RNA from striatal tissue showing the sensitivity of pDYN primers (149 bp). ***C***, Serial dilution of RNA from striatal tissue showing the sensitivity of pENK primers (220 bp). ***D***, A second round of amplification is sufficient to allow for proenkephalin detection in samples containing 1 pg/µl of RNA.

**Figure 3.**
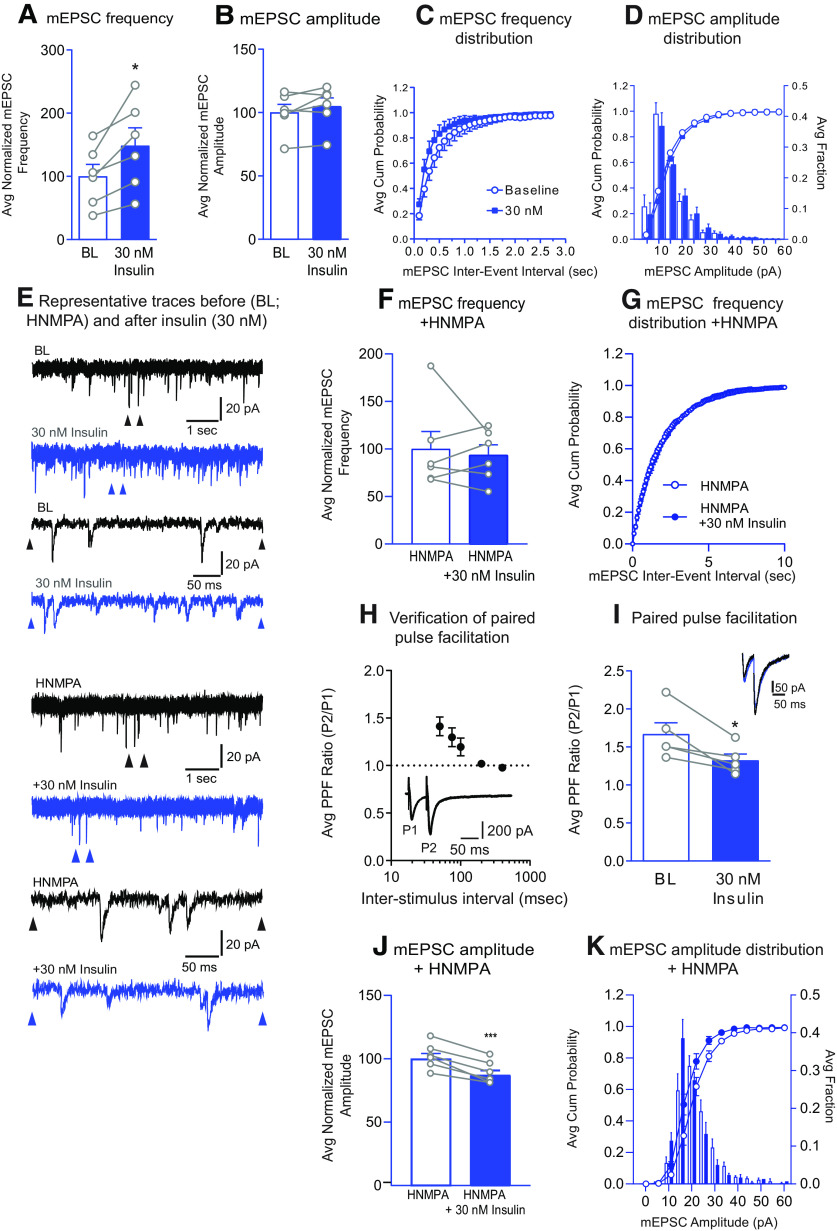
Insulin (30 nm) increases mEPSC frequency and the probability of glutamate release, but not mEPSC amplitude. ***A***, Average mEPSC frequency before (BL) and after bath application of insulin (30 nm). ***B***, Average mEPSC amplitude before and after insulin (30 nm). ***C***, Cumulative probability distributions of mEPSC frequency before and after insulin (30 nm). ***D***, Cumulative probability distributions and histograms of mEPSC amplitude before and after insulin (30 nm). ***E***, Representative mEPSC traces before and after insulin (30 nm), with and without the membrane-impermeable insulin receptor inhibitor HNMPA included in the patch pipette. Top traces, arrows indicate regions in which the time scale was expanded in the lower traces. ***F***, Average mEPSC frequency before and after bath application of insulin (30 nm) with membrane-impermeable HNMPA included in the patch pipette. ***G***, Cumulative probability distributions of mEPSC frequency before and after insulin (30 nm) with HNMPA included in the patch pipette. ***H***, Verification of paired-pulse facilitation in MSNs. Average PPR across increasing interpulse intervals (50-400 ms). Inset, Representative traces at a 50 ms interpulse interval. As expected, the probability of glutamate release is relatively low in NAc MSNs, and facilitation occurs at interpulse intervals ≤100 ms. ***I***, Average PPR before and after insulin (30 nm). Inset, Representative traces before (black) and after insulin (gray) (50 ms interstimulus interval). ***J***, Average mEPSC amplitude before and after insulin (30 nm) with membrane-impermeable HNMPA included in the patch pipette. ***K***, Cumulative probability distributions and histograms of mEPSC amplitude before and after insulin (30 nm) with HNMPA included in the patch pipette. Statistical differences determined by two-tailed paired t-tests, *p < 0.05, ***p < 0.007.

**Figure 4.**
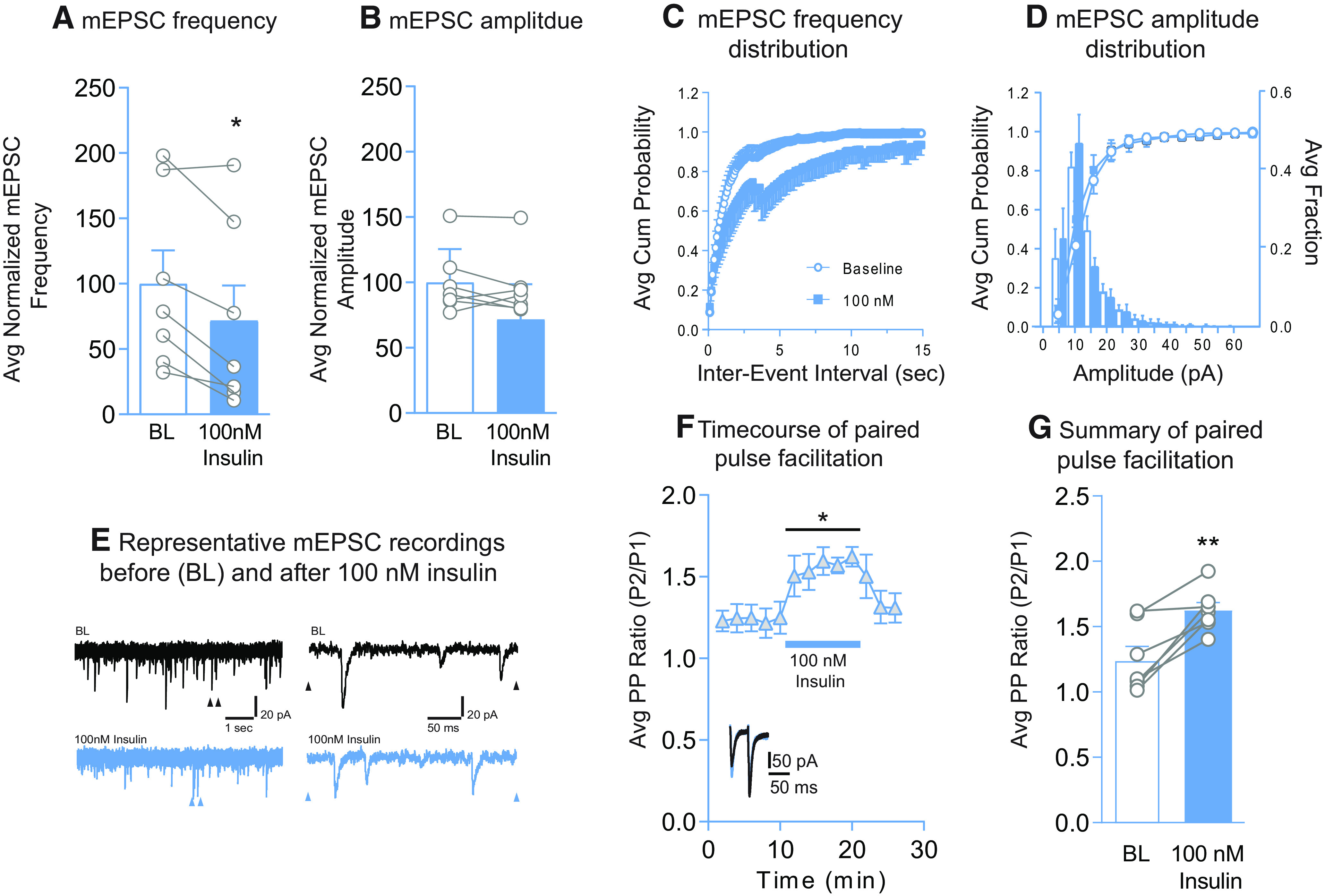
Insulin (100 nm) reduces mEPSC frequency and the probability of glutamate release without altering mEPSC amplitude. ***A***, Average mEPSC frequency before (BL) and after insulin (100 nm). ***B***, Average mEPSC amplitude before and after insulin (100 nm). ***C***, Cumulative probability distributions of mEPSC frequency before and after insulin (100 nm). ***D***, Cumulative probability distributions and histograms of mEPSC amplitude before and after insulin (100 nm). ***E***, Representative mEPSC traces before and after insulin (100 nm). Left traces, arrows indicate regions in which the time scale was expanded in traces shown at the right. ***F***, Average PPR before and after insulin (blue bar, 100 nm) and following insulin washout. Inset, representative traces before (black) and after insulin (blue). ***G***, Average change in the PPR following insulin (100 nm). Statistical differences were determined by two-tailed paired *t* tests (***A***,***G***; **p < 0.01) and two-way repeated-measures ANOVA comparing BL and treatment conditions (***F***; *main effect of treatment, *p* < 0.01).

**Figure 5.**
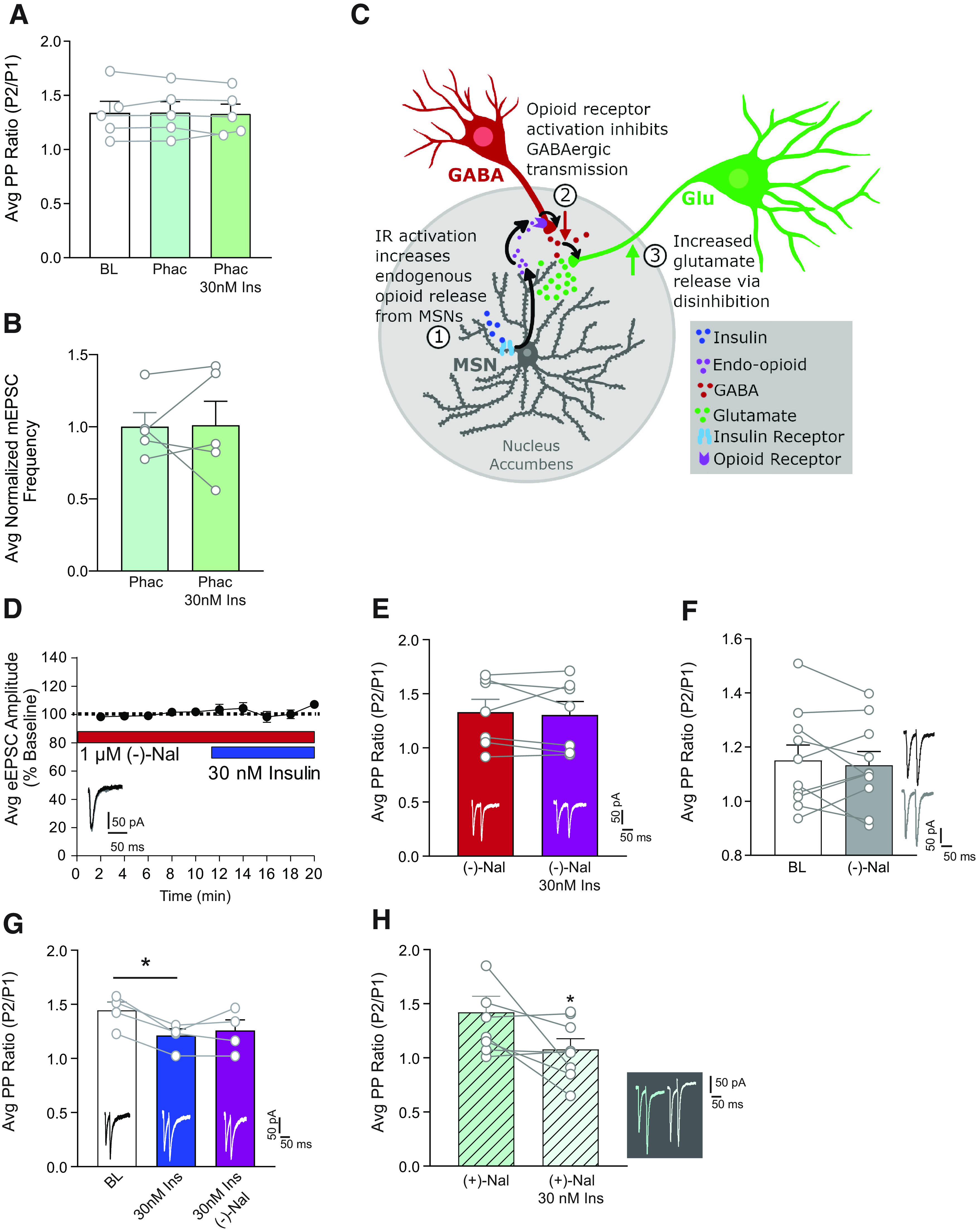
Insulin-induced increases in glutamate release are GABA-B receptor- and opioid-receptor dependent. ***A***, Average PPR at BL, and after insulin (30 nm) in the absence and presence of the GABA_B_ receptor antagonist phaclofen (20 μm). Phaclofen prevents insulin-induced decreases in PPR. ***B***, Average mEPSC frequency before and after insulin (30 nm) in the presence of phaclofen (20 μm). Phaclofen prevents insulin-induced increases in mEPSC frequency. ***C***, Proposed mechanism by which activation of insulin receptors on MSNs enhances glutamate release. We propose that activation of insulin receptors on MSNs results in the release of endogenous opioids (1) that reduces GABAergic transmission (2), thereby causing disinhibition of presynaptic glutamate release (3). ***D***, Average eEPSC amplitude in the presence of the opioid receptor antagonist (–)-naloxone (1 μm) before and after bath application of insulin (30 nm). (–)-Naloxone prevents insulin-induced increases in eEPSC amplitude. ***E***, Average PPR in the presence of (–)-naloxone (1 μm) before and after bath application of insulin (30 nm). (–)-Naloxone prevents insulin-induced decreases in PPR. ***F***, Average PPR before and after bath application of (–)-naloxone (1 μm) alone confirms there is no effect of (–)-naloxone alone. ***G***, Average PPR at BL, and after insulin (30 nm) in the absence or presence of (–)-naloxone (1 μm). (–)-Naloxone does not reverse insulin-induced decreases in PPR. ***H***, Average PPR in the presence of (+)-naloxone (1 μm) before and after bath application of insulin (30 nm). (+)-Naloxone, which does not inhibit opioid receptors, does prevent insulin-induced decreases in PPR. Example traces are shown within each panel. Statistical differences were determined by two-way repeated-measures ANOVA comparing BL and treatment conditions (***D***), two-tailed paired *t* tests (***C***,***E***,***F***,***H***), and one-way ANOVA followed by Sidak's multiple comparisons post-test (***B***,***G***). *p < 0.05.

MSNs can be subdivided into to populations based on their expression of D1- and D2-like receptors that have dissociable roles in motivated behavior (Kravitz et al., 2012; Lenz and Lobo, 2013; R. J. Smith et al., 2013). Within the NAc, D1-type MSNs project to the substantia nigra and VTA (output nuclei), whereas D2- and D1-MSNs project to the ventral pallidum, which is a relay as well as an output nucleus. Compared with studies in the dorsal portion of the striatum, relatively little is known about potential differences in the regulation of excitatory transmission onto D1- versus D2-MSNs. Therefore, as a first step toward examining potential differences in insulin's effects on these two populations, we established single-cell RT-PCR approaches following whole-cell patch clamping to classify a subset of neurons as D1- or D2- type MSNs (cells from data in Figs. 1, 3–5). D2-MSNs were identified by the presence of proenkephalin (pENK) and absence of prodynorphin (pDYN), whereas D1-MSNs were identified by the opposite pattern (Fig. 2*A*). We also determined that the sensitivity of pENK primers was lower than that of pDYN primers (compare Fig. 2*B*,*C*), and that sensitivity of pENK primers can be enhanced by additional amplification (Fig. 2*D*). Of the 72 cells collected, 13 cells could not be classified because bands were not visible (likely due to low starting RNA content). Of the cells identified, 41% were D2-MSNs and 47% were D1-MSNs, consistent with the literature (Sun et al., 2008). The remaining 12% were positive for both pENK and pDYN. This dual expression could be due to contamination from other cells as the pipette was removed from the slice. While this single-cell RT-PCR method can distinguish D1- and D2-MSNs, statistical comparisons were not possible due to low *N* within a given measure in the current study (in part due to starting RNA content, and inability to collect cell contents from all cells). Thus, future studies using transgenic rats specifically designed to identify MSN subpopulations (Pettibone et al., 2019) are needed to make strong conclusions about potential cell type-specific effects (see also Discussion).

### Insulin-induced changes in excitatory transmission are mediated by alterations in presynaptic glutamate release

In Figure 1, we show that insulin bidirectionally alters excitatory transmission. In order to further understand this mechanism, we determined whether these changes in excitatory transmission were due to changes in presynaptic release or alterations in postsynaptic glutamate receptor transmission. Thus, we next examined the effect of 30 or 100 nm insulin on mEPSC amplitude and frequency, as well as on the PPR (Figs. 3, 4). Thirty nM insulin insulin increased mEPSC frequency (Fig. 3*A*; two-tailed paired *t* test: *t*_(5)_ = 3.45, *p* = 0.02; *N* = 6,5) without altering mEPSC amplitude (Fig. 3*B*; two-tailed paired *t* test: *t*_(5)_ = 1.66, *p* = 0.16). In addition, the frequency cumulative probability distribution was shifted to the left compared with BL (Fig. 3*C*), with no change in the amplitude cumulative probability distribution (Fig. 3*D*). These data suggest that insulin-induced increases in excitatory transmission are mediated by enhanced presynaptic glutamate release because we see a change in frequency with no change in amplitude. Furthermore, including HNMPA in the recording pipette blocked this insulin-induced increase in mEPSC frequency (Fig. 3*F*; two-tailed paired *t* test: *t*_(5)_ = 0.465, *p* = 0.66, *N* = 6,3) and prevented the leftward shift in the frequency cumulative probability distribution (Fig. 3*G*), once again confirming that effects are due to activation of postsynaptic insulin receptors on the recorded MSN. Together, these data suggest that increases in presynaptic glutamate release are triggered by activation of MSN-insulin receptors, resulting in feedback from the MSN to presynaptic terminals. To further confirm effects on presynaptic release, we also determined the PPR. We first verified the paired-pulse method in our hands by measuring eEPSCs across a range of interpulse intervals (50, 75, 100, 200, and 400 ms; 6-8 pulses per interval) in the same set of cells. As expected, facilitation occurs at or below an interpulse interval of 100 ms (Fig. 3*H*); thus, an interval of 50 ms was used to test the effect of insulin on PPR. Thirty nM insulin decreased the PPR (Fig. 3*I*; two-tailed paired *t* test: *t*_(4)_ = 3.32, *p* = 0.03; *N* = 5,4), indicative of an increase in the probability of glutamate release; this is consistent with the observed increase in mEPSC frequency (Fig. 3*A*). In addition, when HNMPA was included in the recording pipette and 30 nm insulin was applied, a decrease in mEPSC amplitude was found (Fig. 3*J*; two-tailed paired *t* test: *t*_(5)_ = 7.75, *p* = 0.006, *N* = 6,3) and the cumulative probability distribution of mEPSC amplitudes was shifted to the left (Fig. 3*K*). This suggests additional effects of insulin on postsynaptic transmission that are not mediated by insulin receptor activation.

Effects of 100 nm insulin on mEPSC amplitude and frequency (Fig. 4*A–E*), and paired-pulse facilitation (Fig. 4*F*,*G*) were also examined. We found that the frequency of mEPSCs was significantly reduced by 100 nm insulin (Fig. 4*A*; two-tailed paired *t* test: *t*_(6)_ = 3.90, *p* = 0.008; *N* = 7,6), without altering mEPSC amplitude (Fig. 4*B*; two-tailed paired *t* test: *t*_(6)_ = 0.94, *p* = 0.38). In addition, the frequency cumulative probability distribution was shifted to the right (Fig. 4*C*), with no change in the amplitude cumulative probability distribution (Fig. 4*D*). Consistent with reductions in mEPSC frequency, 100 nm insulin significantly increased the PPR (Fig. 4*F*; two-way repeated-measures ANOVA main effect 100 nm: *F*_(1,6)_ = 21.41, *p* = 0.003; Fig. 4*G*; two-tailed paired *t* test: *t*_(6)_ = 4.67, *p* = 0.003; *N* = 7,5), indicating a reduction in the probability of glutamate release following 100 nm insulin. Thus, activation of IGFRs by insulin reduces excitatory transmission in the NAc core by decreasing glutamate release onto MSNs.

### Insulin-induced increases in excitatory transmission are due to opioid receptor-dependent disinhibition

Endogenous concentrations of insulin in the brain are thought to range from 30 to 50 nm (Havrankova et al., 1978; Schulingkamp et al., 2000). Therefore, we focused studies of underlying mechanisms on insulin receptor-mediated increases in excitatory transmission following 30 nm insulin. Because blockade of insulin receptors within the recorded MSN was sufficient to prevent increases in excitatory transmission resulting from increased presynaptic glutamate release (Figs. 1*D*, 3*F*), we reasoned that presynaptic effects are likely mediated by a neuromodulator released by MSNs, such as GABA or endogenous opioids. Given that both of these transmitters are inhibitory, it is unlikely that effects of insulin are the result of direct effects on presynaptic glutamatergic terminals, as activation of GABA or opioid receptors on glutamatergic terminals reduces presynaptic glutamate release, not enhances it (Nisenbaum et al., 1993; Hjelmstad and Fields, 2003). Therefore, we hypothesized that effects may be due to disinhibition of inhibitory inputs onto glutamatergic terminals (GABA_A_, but not GABA_B_, receptors were blocked during our recordings). Consistent with our hypothesis, addition of the GABA_B_ receptor antagonist phaclofen (20 μm) to the bath was sufficient to prevent insulin-induced increases in glutamate release measured using both PPR (Fig. 5*A*; one-way repeated-measures ANOVA: no effect of 30 nm insulin: *F*_(2,8)_ = 0.074, *p* = 0.93, *N* = 5,3) and mEPSC frequency (Fig. 5*B*; two-tailed paired *t* test: *t*_(5)_ = 0.082, *p* = 0.94, *N* = 5,3). Thus, insulin-induced increases in excitatory transmission appear to rely on disinhibition, rather than direct enhancement of glutamate release. Importantly, the concentration of phaclofen used did not affect basal transmission, suggesting that we did not simply enhance excitatory transmission to a ceiling (data not shown; two-way repeated-measures ANOVA: no effect of 20 μm phaclofen: *F*_(1,10)_ = 2.58, *p* = 0.14, *N* = 6,5). GABA_B_ receptors are widely expressed; therefore, phaclofen will act at both presynaptic and postsynaptic receptors on both glutamatergic and GABAergic neurons, perhaps explaining the lack of an effect of phaclofen alone. However, changes that occur downstream of insulin receptors may be less ubiquitous and instead occur in a specified microcircuit, resulting in the observed increase in excitatory transmission.

Opioid receptors are found on GABAergic terminals in the NAc (Pickel et al., 2004), and activation of opioid receptors causes disinhibition in the VTA and hippocampus by reducing GABAergic transmission (Capogna et al., 1993; Hjelmstad et al., 2013). Thus, we speculated that insulin may trigger endogenous opioid release, which then activates opioid receptors on GABAergic terminals within the NAc to enhance presynaptic glutamate release (see Fig. 5*C*; recordings were done in coronal slices, which contain cell bodies of cells intrinsic to the NAc, and terminals, but not cell bodies, from regions that project to the NAc). Therefore, we next determined whether application of the opioid receptor antagonist (–)-naloxone (1 μm) (Chieng and Christie, 1994) would prevent insulin-induced increases in excitatory transmission. Bath application of (–)-naloxone before 30 nm insulin prevented insulin-induced increases in eEPSC amplitude (Fig. 5*D*; two-way repeated-measures ANOVA no effect of 30 nm insulin: *F*_(1,5)_ = 0.82, *p* = 0.41; *N* = 6,3), and insulin-induced reductions in PPR (Fig. 5*E*; two-tailed paired *t* test: *t*_(6)_ = 0.43, *p* = 0.68; *N* = 7,4). Thus, effects of insulin rely on opioid receptor activation. In addition, (–)-naloxone alone did not alter glutamate release in the absence of insulin (Fig. 5*F*; two-tailed paired *t* test: *t*_(9)_ = 0.53, *p* = 0.53; *N* = 10,5). This suggests that opioid receptor activation is secondary to insulin receptor activation, consistent with the proposed microcircuit shown in Figure 5*C*. Given these results, it is logical to suspect that enhancing basal opioid tone could partially occlude insulin's effects. In an attempt to address this possibility, we used the peptidase inhibitors bestatin (10 μm) and thiorphan (1 μm), which can prevent the degradation of endogenous opioids (Birdsong et al., 2019). Bath application of these peptidase inhibitors decreased eEPSC amplitude from BL, with no further changes observed when 30 nm insulin was applied (data not shown; two-way repeated-measures ANOVA main effect of treatment: *F*_(2,6)_ = 33.2, *p* < 0.001; *N* = 4,3; Sidak's multiple comparisons post-test: BL vs peptidase inhibitors, *p* = 0.003; peptidase inhibitors vs 30 nm, *p* = 0.31). The large reduction in eEPSC amplitude observed when the peptidase inhibitors were bath-applied is consistent with a generalized inhibitory effect of increasing opioid tone but complicates the interpretation of subsequent insulin application. Thus, although the effect of insulin was occluded in these recording conditions, consistent with data above, this could merely be due to the overall inhibition caused by enhancing opioid tone. Interestingly, application of (–)-naloxone after 30 nm insulin was not sufficient to reverse insulin-induced decreases in PPR (Fig. 5*G*; two-way repeated-measures ANOVA main effect of treatment: *F*_(2,6)_ = 6.61, *p* = 0.03; *N* = 4,3; Sidak's multiple comparisons post-test BL vs 30 nm, *p* = 0.04). This is consistent with the absence of effects of (–)-naloxone alone and suggests that, once opioid receptor signaling is triggered, subsequent opioid receptor blockade cannot overcome ongoing signaling. Finally, to more conclusively test the role of opioid receptor activation, we bath-applied (+)-naloxone (1 μm) before 30 nm insulin. (+)-Naloxone is the structural enantiomer of (–)-naloxone but does not have any action at opioid receptors (Iijima et al., 1978). Consistent with the data above, (+)-naloxone did not prevent insulin-induced increases in glutamate release measured by paired-pulse facilitation (Fig. 5*H*; two-tailed paired *t* test: *t*_(7)_ = 2.55, *p* = 0.04; *N* = 8,5). Together, antagonist studies using (–) and (+)-naloxone show that insulin-induced increases in presynaptic glutamate release require opioid receptor activation.

### Diet-induced obesity blunts insulin receptor-mediated increases in excitatory transmission and reduces NAc insulin receptor surface expression

**Figure 6.**
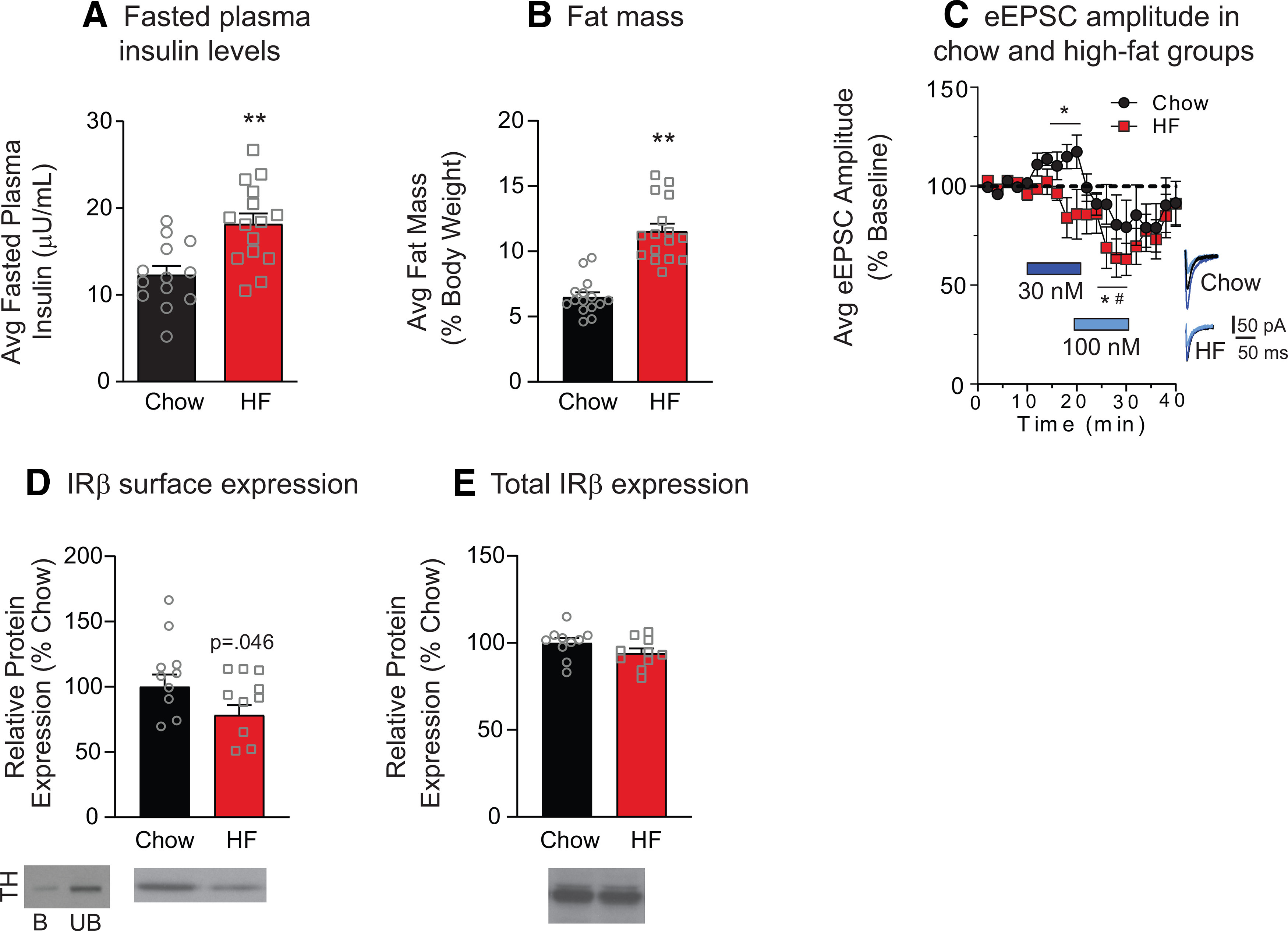
High-fat diet-induced obesity results in a loss of insulin-induced increases in excitatory transmission and a reduction in NAc IRβ surface expression. Average concentration of fasted plasma insulin (***A***) and fat mass (***B***) in chow and high-fat diet groups. ***C***, Average eEPSC amplitude following bath application of increasing concentrations of insulin (blue bars) and following insulin washout in MSNs from chow (circles) and high-fat groups (squares). Right, Representative traces for each group before (black) and after (blue) each insulin concentration. ***D***, Average NAc IRβ surface expression in high-fat and chow-fed groups. Left, immunoblot for TH in the bound (***B***) and unbound (UB) fractions. Consistent with its intracellular localization, TH protein levels were nearly absent in the bound (surface) fraction. ***E***, Total NAc IRβ expression in high-fat and chow-fed groups. Representative blot images are shown below each graph. Statistical differences were determined by two-tailed unpaired *t* tests (***A***,***B***: **p < 0.001), two-way repeated-measures ANOVA comparing BL and treatment conditions (***C***: *chow group, main effect of treatment; ^#^high-fat group, main effect of treatment, *p* < 0.05), and two-tailed unpaired *t* test (***D***: *p* = 0.046).

Circulating insulin reaches the striatum and NAc specifically, and diet-induced obesity is accompanied by chronic elevations in circulating insulin (Woods et al., 2016). In addition, obesity is associated with a reduction in the cognitive-enhancing effects of intranasal insulin in humans (for review, see Kullmann et al., 2016) and impairments in hippocampal glutamatergic plasticity (Fadel and Reagan, 2016). Therefore, we predicted that high-fat diet-induced obesity may blunt insulin's ability to enhance NAc excitatory transmission. For this set of studies, adult male rats were given free access to 60% high-fat diet in the home cage for a total of 8 weeks, whereas controls had free access to standard lab chow. As expected, high-fat diet resulted in significant increases in fasted plasma insulin levels (Fig. 6*A*; two-tailed unpaired *t* test: *t*_(26)_ = 3.65, *p* = 0.001; chow *N* = 13, high-fat *N* = 15) and fat mass compared to controls (Fig. 6*B*; two-tailed unpaired *t* test: *t*_(26)_ = 6.82, *p* < 0.0001). We next examined the effect of bath application of 30 and 100 nm insulin on eEPSC amplitude in slices from high-fat diet and control rats. Similar to results above, 30 nm insulin increased eEPSC amplitude, whereas 100 nm insulin decreased it in recordings from controls (Fig. 6*C*, circles; two-way repeated-measures ANOVA main effect insulin: *F*_(2,8)_ = 5.17, *p* = 0.04; *N* = 5,3). In contrast, in MSNs from high-fat rats, 30 nm insulin did not significantly alter eEPSC amplitude, while significant decreases induced by 100 nm insulin persisted (Fig. 6*C*, squares; two-way repeated-measures ANOVA main effect insulin: *F*_(2,10)_ = 8.74, *p* = 0.01; BL vs 30 nm; main effect of insulin: *F*_(1,5)_ = 2.86, *p* = 0.15; BL vs 100 nm; main effect of insulin: *F*_(1,5)_ = 8.58, *p* = 0.03; *N* = 6,5). This occlusion of insulin-induced increases in the high-fat group suggest physiological roles for insulin in the NAc, and are consistent with the idea that physiological shifts in circulating insulin secondary to diet-induced obesity impact neural insulin sensitivity (Ferrario and Reagan, 2018).

One potential explanation for the loss of insulin-induced increases in excitatory transmission is a reduction in NAc insulin receptor expression. Therefore, we next determined the effect of high-fat diet on surface expression of the obligatory β subunit (IRβ) of the insulin receptor using established biotinylation and pulldown procedures (Ferrario et al., 2011). The intracellular protein TH was apparent in the unbound, but not the bound, fraction as expected (Fig. 6*D*). In NAc tissue from chow and high-fat groups, we found a 21.7% (±7.6%) reduction in IRβ surface expression in high-fat versus chow-fed groups (Fig. 6*D*; two-tailed unpaired *t* test: *t*_(18)_ = 1.75, *p* = 0.046) without any changes in total IRβ (Fig. 6*E*). This suggests that reductions in NAc insulin receptor expression may contribute to the loss of insulin-induced increases in eEPSC amplitude in the high-fat group.

## Discussion

### Bidirectional effects of insulin receptor and IGFR activation

We found bidirectional effects of insulin on NAc excitatory transmission, with 30 nm insulin increasing, and 100-500 nm decreasing, eEPSC amplitude (Fig. 1*A*,*B*). Using antagonists, we show that increases in excitatory transmission are mediated by insulin receptors, whereas decreases are mediated by IGFRs. Furthermore, insulin receptor-mediated effects were attributable to activation of insulin receptors on MSNs, as adding a membrane-impermeable insulin receptor blocker in the recording pipette completely prevented insulin-induced increases in excitatory transmission. The magnitude of the insulin-induced increase in eEPSC amplitude was similar in the presence or absence of an IGFR antagonist (∼25%-30%). This suggests that 30 nm insulin only activates insulin receptors, whereas higher concentrations are required to recruit IGFR activation. Indeed, 50 nm insulin did not alter excitatory transmission, presumably because the sum of enhancing (insulin receptor-mediated) and inhibitory (IGFR-mediated) effects were off-setting (Fig. 1*A*). These results suggest that the net effect of insulin on excitatory transmission *in vivo* may be influenced by local insulin concentration (see also, effects of high-fat below).

We next determined whether effects on excitatory transmission are due to alterations in presynaptic or postsynaptic function. Application of 30 nm insulin increased mEPSC frequency without altering mEPSC amplitude, an effect that was blocked by preventing insulin receptor signaling (Fig. 3*A*,*B*,*F*). This same concentration of insulin also enhanced the probability of glutamate release (Fig. 3*I*). Thus, reductions in the PPR in combination with increases in mEPSC frequency strongly support insulin-induced enhancement of glutamate release. To our knowledge, this is the first time insulin has been found to enhance glutamate release. In contrast, in VTA, insulin receptor activation produces rapid and persistent reductions in presynaptic glutamate release (Labouebe et al., 2013; Liu et al., 2013). Thus, effects of insulin appear to be region-specific, although studies in VTA were conducted in cultured neurons or juvenile mice, while studies here are in adult rats. IGFR-mediated reductions in excitatory transmission were also due to effects on presynaptic glutamate release (Fig. 4). This is consistent with the ability of IGFR activation to inhibit L-type calcium channel activity, which mediates presynaptic glutamate release (Subramanian et al., 2013; Sánchez et al., 2014), and with the suppression of spontaneous excitatory transmission in hippocampus by IGFR activation (Gazit et al., 2016).

### How does postsynaptic insulin receptor activation result in increased presynaptic glutamate release?

Blockade of insulin receptor signaling within the recorded MSN was sufficient to prevent insulin-induced increases in excitatory transmission (Figs. 1*D*, 3*F*), suggesting a mechanism involving feedback from MSNs to presynaptic glutamatergic terminals. Given that transmitters released by MSNs are inhibitory, we hypothesized that effects may be due to disinhibition of inhibitory inputs onto glutamatergic terminals. Indeed, our data support a previously unidentified mechanism whereby insulin produces disinhibition that is dependent on opioid receptor activation (Fig. 5).

Recordings were made in the presence of a GABA_A_ antagonist; thus, ionotropic inhibition cannot contribute. Addition of a GABA_B_ antagonist prevented insulin-induced increases in release probability and mEPSC frequency (Fig. 5*A*,*B*). While removing all GABA transmission is quite a “hammer,” this nonetheless provides additional support for disinhibition. Addition of the opioid receptor antagonist (–)-naloxone was sufficient to prevent insulin-induced increases in excitatory transmission measured by mEPSC frequency, PPR, and eEPSC amplitude (Fig. 5*A–E*). The role of opioid receptors was further supported by the inability of (+)-naloxone (which does not have any action at opioid receptors) (Iijima et al., 1978) to prevent insulin-induced increases in glutamate release (Fig. 5*H*). Additionally, naloxone alone was not sufficient to alter glutamate release (Fig. 5*F*), suggesting that there is not opioid-dependent tonic inhibition of presynaptic glutamate. This may be due to rapid degradation of endogenous opioids by peptidases (see also below). However, tonic inhibition is not necessarily required for the observed effects of insulin. Rather, we propose that activation of insulin receptors on MSNs leads to elevations in endogenous opioids, thereby causing disinhibition of presynaptic glutamate release (Fig. 5*C*).

Although few functional studies have examined the regulation of NAc glutamate release by endogenous opioids, this mechanism is consistent with anatomic and physiological data. Specifically, mu opioid receptors are located on presynaptic GABAergic terminals within the NAc (Svingos et al., 1997; Pickel et al., 2004), and mu opioid receptor activation reduces GABA release in the hippocampus and subthalamic nucleus (Lambert et al., 1991; Xie et al., 1992; Capogna et al., 1993; Lupica, 1995; Shen and Johnson, 2002). In addition, GABA_B_ receptors are located on glutamatergic terminals within the striatum where they inhibit excitatory transmission (Nisenbaum et al., 1993). Thus, it is feasible for endogenous opioids to produce the disinhibition observed here (Shen and Johnson, 2002; Banghart et al., 2015; Tejeda et al., 2017).

Naloxone is a nonselective opioid receptor antagonist. Kappa opioid receptors are located on terminals of excitatory and inhibitory synapses within the NAc (Svingos et al., 1999; Meshul and McGinty, 2000; Tejeda et al., 2017), and on dopamine afferents in the NAc (Spanagel et al., 1992), whereas delta opioid receptors are preferentially expressed on cholinergic interneurons within the NAc (Le Merrer et al., 2009; Bertran-Gonzalez et al., 2013; Castro and Bruchas, 2019). Thus, in addition to potential roles for mu opioid receptors discussed above, effects could be mediated by one, or a combination of different opioid receptors. Future studies are needed to determine the receptor population(s) involved.

In the course of the studies above, we found that inclusion of peptidase inhibitors reduced eEPSC amplitude by ∼28% (±4.3). This suggests that, under these conditions, there may be an accumulation of endogenous opioids within our slices. Although additional studies are needed to confirm this observation, these data are consistent with one previous report examining dorsal striatum (Atwood et al., 2014; but for additional discussion, see Birdsong and Williams, 2020). Overall, reductions in eEPSC amplitude following peptidase inhibition provide indirect evidence for accumulation of endogenous opioids that warrants future study.

Finally, while studies using transgenics are needed to draw firm conclusions about potential heterogeneity of effects across D1- and D2-type MSNs, the consistency of insulin's effect across recorded cells suggests that potential differences may be subtle. That is, if the effects of insulin were isolated to one population, or were opposite in the two populations, one would expect that recording from both cell types indiscriminately would result in null effects. But this is not the case; instead, we find consistent effects of insulin across different measures. This may not be entirely surprising given that the endogenous opioids dynorphin A17 (from D2-MSNs) and enkephalins (met-enkephalin and leu-enkephalin; from D1-MSNs) have activity at kappa, delta, and mu opioid receptors found within the striatum (e.g., Gomes et al., 2020; and references therein).

### Loss of insulin-receptor mediated effects following obesity

When effects of high-fat diet were examined, we found a loss of insulin receptor-mediated increases in excitatory transmission, but a maintenance of IGFR-mediated decreases (Fig. 6*C*). Although slight trends were seen for reduced transmission following 30 nm insulin in the high-fat group, the *p* value indicated a low probability of a true effect. The loss of insulin-induced increases may be due in part to modest reductions in NAc insulin receptor expression, as surface expression of IRβ was reduced following high-fat diet (Fig. 6*D*). However, concomitant reductions in signaling downstream of the receptor could also contribute. This is an avenue for future investigation. A reduction in insulin receptor expression is consistent with the development of insulin resistance in the face of chronic elevations in circulating insulin resulting from obesity, and with impairments in hippocampal glutamatergic plasticity induced by insulin resistance (Fadel and Reagan, 2016). Although we cannot rule out the contribution of differences in basal insulin tone between chow and high-fat groups, these data nonetheless demonstrate that physiologically relevant increases in circulating insulin are accompanied by reductions in insulin receptor-induced effects on NAc excitatory transmission.

In conclusion, studies above provide the first insights into how insulin influences NAc excitatory transmission. Based on these results, we propose that activation of insulin receptors on MSNs results in enhanced activity of endogenous opioids, ultimately producing disinhibition of presynaptic glutamate release (Fig. 5*C*). In addition, data show that insulin receptors and IGFRs work in opposition to enhance and reduce glutamatergic transmission, respectively. This, in combination with recordings from obese rats, strongly suggests that shifts in the balance of activity at these receptors will influence the ability of insulin to regulate NAc activity. Thus, future studies will be needed to determine how insulin may affect motivation and feeding-related processes in the obese and nonobese state that are mediated by NAc excitatory transmission and endogenous opioids (Zhang and Kelley, 2000; Richard et al., 2013; Katsuura and Taha, 2014; Castro and Bruchas, 2019; Ferrario, 2020). Finally, the NAc receives inhibitory input from GABAergic neurons in the VTA (Van Bockstaele and Pickel, 1995), local collateralization of MSNs, and aspiny GABAergic interneurons (A. D. Smith and Bolam, 1990; Kawaguchi, 1993; Planert et al., 2010). Thus, in addition to identifying the opioid receptors involved, it will be important for future studies to determine whether disinhibition produced by insulin is selective to different sources of GABA within the NAc.
